# Seedability: optimizing alignment parameters for sensitive sequence comparison

**DOI:** 10.1093/bioadv/vbad108

**Published:** 2023-08-12

**Authors:** Lorraine A K Ayad, Rayan Chikhi, Solon P Pissis

**Affiliations:** Department of Computer Science, Brunel University London, London UB8 3PH, UK; G5 Sequence Bioinformatics, Institut Pasteur, Université Paris Cité, 75015 Paris, France; Networks & Optimization, CWI, 1098 XG Amsterdam, The Netherlands; Department of Computer Science, Vrije Universiteit, 1081 HV Amsterdam, The Netherlands

## Abstract

**Motivation:**

Most sequence alignment techniques make use of exact *k*-mer hits, called seeds, as anchors to optimize alignment speed. A large number of bioinformatics tools employing seed-based alignment techniques, such as Minimap2, use a single value of *k* per sequencing technology, without a strong guarantee that this is the best possible value. Given the ubiquity of sequence alignment, identifying values of *k* that lead to more sensitive alignments is thus an important task. To aid this, we present Seedability, a seed-based alignment framework designed for estimating an optimal seed *k*-mer length (as well as a minimal number of shared seeds) based on a given alignment identity threshold. In particular, we were motivated to make Minimap2 more sensitive in the pairwise alignment of short sequences.

**Results:**

The experimental results herein show improved alignments of short and divergent sequences when using the parameter values determined by Seedability in comparison to the default values of Minimap2. We also show several cases of pairs of real divergent sequences, where the default parameter values of Minimap2 yield no output alignments, but the values output by Seedability produce plausible alignments.

**Availability and implementation:**

https://github.com/lorrainea/Seedability (distributed under GPL v3.0).

## 1 Introduction

Comparing genomic sequences is essential for genome-wide analyses, such as phylogenetic inference, genome annotation, and function prediction (Dewey 2012). Similarly, aligning protein sequences is required for template-based protein structure prediction and function annotation (Yan *et al.* 2013). Traditional techniques for global sequence alignments (Needleman and Wunsch 1970, Gotoh 1982), where entire sequences are to be compared, commonly use dynamic programming, which can be inefficient for very long sequences. This can also be particularly time-consuming when aligning a query sequence to a database of reference sequences, e.g. RefSeq (O’Leary *et al.* 2016).

Seed-based alignment techniques have become increasingly popular, due to their moderate resource requirements, in comparison to the traditional dynamic-programming-based methods, as well as maintaining a high alignment accuracy. Many seed-based techniques make use of *k*-mers (Luczak *et al.* 2019, Alser *et al.* 2021), which are short substrings of fixed length *k*. In a nutshell, when a reference *k*-mer is found within a query sequence, the match is referred to as a *hit* or *seed*. The well-known BLAST software (Altschul *et al.* 1990) uses *k*-mer seeds, which are then chained and extended to produce alignment(s) between target and query sequences. Spaced-seeds (binary patterns of symbols 0 and 1, denoting a match and a wildcard, respectively) have also been used extensively to improve alignment results via higher sensitivity when compared to traditional seed-based techniques (Ma *et al.* 2002). For instance, Khiste and Ilie (2017) employ the notion of spaced-seeds for assembling PacBio data showing higher alignment detection sensitivity in comparison to pre-existing tools. Other less common sequence comparison techniques include employing the notion of longest common substring (Leimeister and Morgenstern 2014) or common absent words (Charalampopoulos *et al.* 2018), or even employing Fourier transformations (Yin and Yau 2015).

Roberts *et al.* (2004) introduced the idea of sampling seeds using minimizers, where only a small fraction of seeds need to be stored during computations. Minimizers can speed up string-matching computations by a large factor while missing only a small fraction of the matches found using all seeds. Intuitively, this is because, when a target sequence exactly matches a query sequence, the same minimizers are sampled from both sequences. Minimap2 (Li 2018) is a versatile sequence alignment program that uses minimizers as seeds to compute alignments of DNA (or mRNA) sequences against a large set of reference sequences. Typical use cases of Minimap2 include, among others, aligning sequence reads to a reference genome or constructing whole-genome alignments between two closely-related species (for instance, with divergence below ∼15%).

Minimap2 uses a default value for the seed length *k*, which can also be specified by the user on input. It should be clear that varying the length of seeds has an impact on the efficiency and the output alignments of Minimap2. For instance, setting a small value for *k* may increase alignment accuracy as it allows more seeds to be identified. However, this comes at the cost of increasing running time due to the increased number of identified seeds that require further processing. On the other hand, setting a large value for the seed length *k* reduces the running time but may result in poorer alignments and even in alignments that are entirely missed. We hypothesize that the performance of any seed-based alignment algorithm can be impacted by tuning the *k* value appropriately. Yet, it is unclear how a user may make an educated guess about setting *k*. Therefore, there is a need for an automated method for identifying appropriate values for *k*.

While optimizing the values for parameter *k* has been studied for genome assembly (Chikhi and Medvedev 2013), optimizing the seed length *k* appears to have only been studied for variants of BLAST (Gotea *et al.* 2003, Shiryev *et al.* 2007). To the best of our knowledge, recent methods for sequence comparison, in particular, Minimap2, have not received the same treatment. To aid this, we present Seedability, a framework designed for computing an optimal *k*-mer length as well as an accurate number of shared seeds between a unique given set of sequences.

In the following, we introduce a theoretical alignment framework and formulate the *Seedability* problem. The problem consists in finding optimal parameter values for an idealized version of seed-based alignment. The precise computational task is, given an alignment identity threshold, to estimate an optimal seed *k*-mer length as well as a minimal number *t* of shared seeds for aligning pairs of sequences in a given collection. One can then combine these parameter values to infer optimal parameter values in different alignment tools based on their underlying alignment mechanism. In particular, we demonstrate that the parameter values found by Seedability can be directly used to tune the alignment parameters for increasing the sensitivity of Minimap2 when aligning pairs of short sequences. We show, among others, that in this new regime, Minimap2 becomes capable of aligning sequences of lengths 200, 300, 500, or 1000 base pairs (bp) with a divergence of 25% with an average alignment success rate improvement of 0.57, 0.65, 0.68, and 0.12 points, respectively, compared to when using its default values with preset option sr.

The paper is organized as follows. In Section 2, we provide the necessary definitions and notation. In Section 3, we present the Seedability framework. In Section 4, we present our results. We conclude in Section 5.

## 2 Definitions and notation

A *string* (or *sequence*) *x* of length |x|=m is an array x[0…m−1], where every x[i], 0≤i<m, is a letter drawn from some fixed alphabet Σ. An *empty string* is the string of length 0, and it is denoted by *ε*. A string *x* is a *substring* (or *fragment*) of string *y* if there exist two strings *u* and *v*, such that *y* = *uxv*. When *x* is a substring of *y*, we say that *x occurs* in *y*. Each occurrence of *x* can be specified by a position in *y*. We say that *x* occurs at (the starting) position *i* in *y* when y[i…i+m−1]=x. A *k-mer*, for any integer *k *>* *0, is a string from $\Sigma^{k}$. For any two strings *x* and *y* and an integer *k *>* *0, we define a *seed* (or *hit*) of *x* and *y*, a pair (*i*, *j*) such that x[i…i+k−1] and y[j…j+k−1] is the same *k*-mer.

Given two strings *x* and *y* and an integer *k *>* *0, we say that *x* and *y share t seeds*, for some integer t≥0, if and only if there exists a sequence $i_{1},\ldots ,i_{t}$ of *t* positions on *x* and a sequence $j_{1},\ldots ,j_{t}$ of *t* positions on *y*, such that all of the following hold: $x[i_{1}\ldots i_{1}+k-1]=y[j_{1}\ldots j_{1}+k-1],\ldots ,x[i_{t}\ldots i_{t}+k-1]=y[j_{t}\ldots j_{t}+k-1]$.

For example, given x=ACGTAGTAG, y=ACGAGTAGG, and *k *=* *3, *x* and *y* share *t *=* *4 seeds. This is because there exists a sequence 0,4,5,6 of positions on *x*, and a sequence 0,3,4,5 of positions on *y* such that x[0…2]=y[0…2]=ACG, x[4…6]=y[3…5]=AGT, x[5…7]=y[4…6]=GTA and x[6…8]=y[5…7]=TAG.

Given a string *x* of length *m* and a string *y* of length *n*, the *Levenshtein distance* (or *edit distance*) (Levenshtein 1965), denoted by $\delta_{L}(x,y)$, is the minimum total number of elementary edit operations required to transform *x* into *y*. In particular, the elementary edit operations we consider are:

- *insertion*: insert a letter of *y* in *x* at a given position;
- *deletion*: delete a letter of *x* at a given position;
- *substitution*: substitute a letter of *x* at a given position by a letter of *y*.

For any two strings *x*, *y*, the distance $\delta_{L}(x,y)$, can be computed in *O*(*mn*) time (Levenshtein 1965). An *alignment* between *x* and *y* is another string *z* on the alphabet of pairs of letters, more accurately on whose projection on the first component is *x* and the projection on the second component is *y*. An insertion in *z* is represented by (ε,a), a∈Σ; a deletion in *z* is represented by (a,ε), a∈Σ; and a substitution in *z* is represented by (*a*, *b*), a,b∈Σ and a≠b. The *cost* of an alignment *z* is the total number of insertions, deletions and substitutions in *z*. In our model, an alignment *z* is *optimal* if and only if its cost is precisely $\delta_{L}(x,y)$. The *alignment identity* $e_{x,y}(z)$ of an alignment *z* of *x* and *y* is defined as where Σsub is the total number of substitutions, Σdel is the total number of deletions, and Σins is the total number of insertions in *z*. The alignment identity is computed by working out as a fraction, the number of matches in the alignment over the alignment length. Note that the alignment length is equal to |x| plus the total number of insertions in *z*. The *divergence* $d_{x,y}(z)$ is the complementary notion and it is equal to $1-e_{x,y}(z)$. When *z* is an optimal alignment of *x*, *y*, we call $e_{x,y}(z)$ and $d_{x,y}(z)$ the *optimal alignment identity* and the *optimal divergence*, respectively.


(Σ∪{ε})×(Σ∪{ε})∖{(ε,ε)},

$$e_{x,y}(z)=\frac{\left|x|-(\Sigma \mathrm{sub}+\Sigma \mathrm{del}\right)}{|x|+\Sigma \mathrm{ins}},$$

Given a string *x* and an integer κ>0, a *minimizer* of *x* is a lexicographically smallest *κ*-mer in *x*. Given a string *x* and two integers κ>0 and *w *>* *0, the set of (κ,w)-minimizers of *x* is the set of positions of minimizers of all length-(w+κ−1) fragments of *x*. If more than one (κ,w)-minimizer exists in one fragment, we can *consistently* sample one of them; e.g. we can always choose the leftmost one as the minimizer.

## 3 Methods

We start by formally defining the computational problem considered here. Let *S* be a set of input sequences. For presentation purposes, we will assume that all sequences in *S* have the same length. In practice, our algorithms will work on sequences that have different but similar lengths. We relate the proposed framework to the classic read-to-reference alignment framework (e.g. of Minimap2), where a set of input reads are to be aligned against several candidate positions of the reference. In such a scenario, one may convert a set *S_d_* of input sequences, where sequences have different lengths, to another set *S*, where all sequences have the same length. For instance, one can create *S* such that it consists of all the length-*W* substrings of the sequences of *S_d_*, where *W* is a chosen window length smaller than or equal to the shortest sequence in *S_d_*. Thus, in the rest of this section, we will assume that all sequences have the same length.

Given *S*, we define the set $O_{\text{truth},e}$ as the set of all pairs of sequences (*s*_1_, *s*_2_), $s_{1},s_{2}\in S$, such that *s*_1_ and *s*_2_ have optimal alignment identity greater than or equal to *e*. We now formally define the problem in scope:

Problem 1 (Seedability). *Given S and an alignment identity threshold e, compute a set* $O_{\text{seed}}$*of pairs of sequences from S and one pair (t, k) of values, for every pair of sequences in* $O_{\text{seed}}$*, such that the symmetric difference of* $O_{\text{seed}}$*and* $O_{\text{truth},e}$*is minimized.*

By estimating a *k*-mer length for every pair of sequences in a given collection for a given alignment identity threshold, we can aggregate these *k* values to infer an optimal (κ,w) value for Minimap2. This is precisely the main application of our alignment framework in this article.

### 3.1 The Seedability algorithm

We propose the following algorithm, which we call Seedability, as a heuristic approach to address Problem 1.

Let *S* be a set $\left\{s_{1},\ldots ,s_{r}\right\}$ of *r* sequences. The Seedability algorithm is a two-stage approach that is carried out for all $\min_{k}\leq k\leq \max_{k}$, where $\left[\min_{k},\max_{k}\right]$ is defined by the user. The default value for $\min_{k}$ is 3. The default value for $\max_{k}$ is 15, which is the default value in Minimap2 for the length *κ* of minimizers. The two stages are:

1. Estimating $e_{s_{i},s_{j}}$, for all $i\neq j\in [1,r],\;s_{i},s_{j}\in S$;
2. Constructing the set $O_{\text{seed}}$.

The main idea of our algorithm is to use *k*-mers to identify seeds shared between the given pair of sequences. We then traverse through the seeds to *estimate* Σins, Σdel, and Σsub, thus *estimating* alignment identity $e_{s_{i},s_{j}}$. Finally, for every pair of sequences (*s_i_*, *s_j_*), we want to output one (*t*, *k*) value for which the corresponding $e_{s_{i},s_{j}}$ exceeds or equates to the alignment identity threshold *e*. If such a (*t*, *k*) value exists, then (*s_i_*, *s_j_*) is added to $O_{\text{seed}}$.

### 3.2 Estimating $e_{s_{i},s_{j}}$

**Figure 1.**
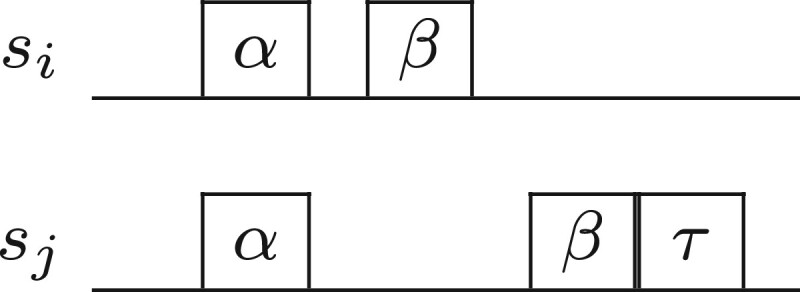
Step 1 of estimating $e_{s_{i},s_{j}}$.

**Figure 2.**
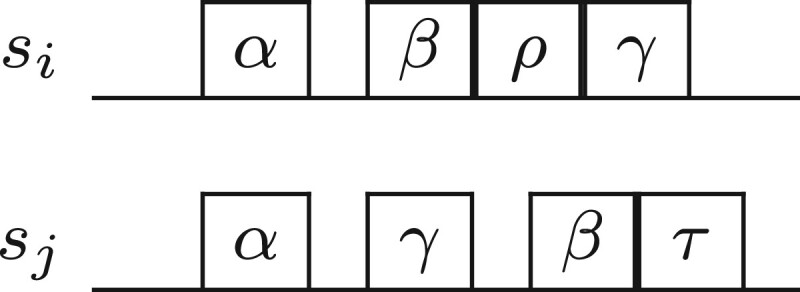
Step 2 of estimating $e_{s_{i},s_{j}}$.

We next present the techniques which we employ to estimate *t*, the number of seeds shared by *s_i_* and *s_j_*, which allows us to then estimate $e_{s_{i},s_{j}}$. Given a seed (*p_i_*, *p_j_*) on the pair of sequences (*s_i_*, *s_j_*), if $s_{i}[p_{i}\ldots 1\ldots p_{i}+k]=s_{j}[p_{j}\ldots 1\ldots p_{j}+k]$, then $\left(p_{i}+1,p_{j}+1\right)$ is the next chosen seed. This can be easily checked in constant time. If $s_{i}[p_{i}\ldots 1\ldots p_{i}+k]\neq s_{j}[p_{j}\ldots 1\ldots p_{j}+k]$, then the following steps are carried out:

1. Let *s_i_* and *s_j_* be two sequences, where $s_{i_{\alpha }}$ is an occurrence of *k*-mer *α* in *s_i_* and $s_{j_{\alpha }}$ an occurrence of the same *k*-mer in *s_j_*. Let us assume that the pair $\left(s_{i_{\alpha }},s_{j_{\alpha }}\right)$ is a previously selected seed. (Note that we can always start with a dummy seed (−1,−1).) Then let $s_{i_{\beta }}$ be the smallest occurrence of some *k*-mer *β* in *s_i_* such that there exists an occurrence $s_{j_{\beta }}$ of the same *k*-mer in *s_j_* with $s_{i_{\beta }}>s_{i_{\alpha }}$ and $s_{j_{\beta }}>s_{j_{\alpha }}$. We find the occurrence of *k*-mer τ=β in *s_j_* (see Fig. 1) such that $\left|(s_{i_{\beta }}-s_{i_{\alpha }})-(s_{j_{\tau }}-s_{j_{\alpha }})\right|$ is minimized and $|(s_{i_{\beta }}-s_{i_{\alpha }})-(s_{j_{\tau }}-s_{j_{\alpha }})|\leq k$. If this holds, for some $s_{j_{\tau }}$, then the occurrences $s_{i_{\beta }}$ and $s_{j_{\tau }}$ form a *candidate seed*. The inequality ensures that the pair of *β* occurrences to be selected as a candidate seed are at a similar distance from the corresponding *α* occurrences. For every $s_{i_{\beta }}$ (we have $O(|s_{i}|)$ of them), this check can be implemented in *O*(*k*) time due to the condition $|(s_{i_{\beta }}-s_{i_{\alpha }})-(s_{j_{\tau }}-s_{j_{\alpha }})|\leq k$. This is because $s_{i_{\beta }},s_{i_{\alpha }},s_{j_{\alpha }}$ are fixed and the only unknown is $s_{j_{\tau }}$.
2. Again, let us assume that the pair $\left(s_{i_{\alpha }},s_{j_{\alpha }}\right)$ is a previously selected seed. Let $s_{j_{\gamma }}$ be the smallest occurrence of some *k*-mer *γ* in *s_j_* such that there exists an occurrence $s_{i_{\gamma }}$ in *s_j_* with $s_{j_{\gamma }}>s_{j_{\alpha }}$ and $s_{i_{\gamma }}>s_{i_{\alpha }}$. We find the occurrence of *k*-mer ρ=γ in *s_i_* (see Fig. 2) such that $\left|(s_{j_{\gamma }}-s_{j_{\alpha }})-(s_{i_{\rho }}-s_{i_{\alpha }})\right|$ is minimized and $|(s_{j_{\gamma }}-s_{j_{\alpha }})-(s_{i_{\rho }}-s_{i_{\alpha }})|\leq k$. If this holds, for some $s_{i_{\rho }}$, then the occurrences $s_{j_{\gamma }}$ and $s_{i_{\rho }}$ form a *candidate seed*. This is precisely the symmetric computation of the first step. For every $s_{j_{\gamma }}$ (we have $O(|s_{j}|)$ of them), this check can be implemented in *O*(*k*) time due to the condition $|(s_{j_{\gamma }}-s_{j_{\alpha }})-(s_{i_{\rho }}-s_{i_{\alpha }})|\leq k$.
3. The two candidate seeds are now compared to select one of them. Let the first one be $\left(s_{i_{\beta }},s_{j_{\tau }}\right)$ and the second one be $\left(s_{i_{\rho }},s_{j_{\gamma }}\right)$. If $\left|(s_{i_{\beta }}-s_{i_{\alpha }})-(s_{j_{\tau }}-s_{j_{\alpha }})|\leq |(s_{i_{\rho }}-s_{i_{\alpha }})-(s_{j_{\gamma }}-s_{j_{\alpha }})\right|$ then $\left(s_{i_{\beta }},s_{j_{\tau }}\right)$ forms the next seed, otherwise $\left(s_{i_{\rho }},s_{j_{\gamma }}\right)$ forms the next seed. We proceed to the computation of the next shared seed (by memorizing the one we have just computed as the new previously selected seed) until no other seed can be selected.

The computation of shared seeds, between every pair of sequences in *S*, using the two steps described above, allows us to estimate the alignment identity as follows. Let $\left(s_{i_{\alpha }},s_{j_{\alpha }}\right)$ and $\left(s_{i_{\beta }},s_{j_{\beta }}\right)$ be two consecutive encountered seeds. When a gap of size less than *k* is encountered between the two seeds (i.e., $|(s_{i_{\beta }}-s_{i_{\alpha }})-(s_{j_{\beta }}-s_{j_{\alpha }})|\leq k$), the number of letters within the gaps in *s_i_* and *s_j_* are added onto Σsub, Σdel or Σins. Specifically, if the size of the gap is the same in *s_i_* and *s_j_*, then Σsub is incremented by the size of the gap. If the size of the gap in *s_i_* is larger than that in *s_j_* then Σsub is incremented by the size of the gap in *s_j_* and Σdel is incremented by the difference in size of the gaps. If the size of the gap in *s_j_* is larger than that in *s_i_* then Σsub is incremented by the size of the gap in *s_i_* and Σins is incremented by the difference in size of the gaps. The computation of Σsub, Σdel, and Σins results in the estimation of $e_{s_{i},s_{j}}$ for the (*t*, *k*) values considered. The total number of shared seeds is $O(|s_{i}|+|s_{j}|)$ and so this computation takes $O(|s_{i}|+|s_{j}|)$ time. Overall, the whole computation takes $O(k(|s_{i}|+|s_{j}|))$ time for any pair $s_{i},s_{j}\in S$ of sequences and any $k\in [\min_{k},\max_{k}]$.

For example, let $s_{i}=\mathrm{GCGTGATTCG},\;s_{j}=\mathrm{GCGGATTGAG}$ and *k *=* *3. Clearly, the first computed seed is $\left(s_{i_{\alpha }},s_{j_{\alpha }})=(0,0\right)$ representing α=GCG. Then, for the second seed, we have two candidates: the first candidate seed is ($s_{i_{\beta }},s_{j_{\tau }})=(3,6)$ representing β=TGA (Step 1); the second candidate seed is $\left(s_{i_{\rho }},s_{j_{\gamma }})=(4,3\right)$ representing γ=GAT (Step 2). The chosen seed is $\left(s_{i_{\rho }},s_{j_{\gamma }})=(4,3\right)$ computed in Step 2.

**Table:** 

Step 1	Step 2
$s_{i}=\underline{\mathrm{GCG}}\underline{\mathrm{TGA}}\mathrm{TTCG}$	$s_{i}=\underline{\mathrm{GCG}}T\underline{\mathrm{GAT}}\mathrm{TCG}$
$s_{j}=\underline{\mathrm{GCG}}\mathrm{GAT}\underline{\mathrm{TGA}}G$	$s_{j}=\underline{\mathrm{GCG}}\underline{\mathrm{GAT}}\mathrm{TGAG}$

Alignments *z*_1_ and *z*_2_ below show the final alignments if the first seed was chosen (*z*_1_) in comparison to if the second seed was chosen (*z*_2_). If the first seed is chosen, there are no further seeds identified in *s_i_* and *s_j_*, and the alignment identity is 6/13. If, however, the second seed is chosen, there is one further seed identified in *s_i_* and *s_j_*, and the alignment identity is 7/11>6/13. In fact, this (*z*_2_) is what our algorithm chooses.

**Table:** 

*z*_1_	*z*_2_
GCG- --TGATTCG	GCGTGATTCG-
GCGGATTGAG---	GCG-GATTGAG

### 3.3 Constructing the set $O_{\text{seed}}$

Recall that we aim at minimizing the symmetric difference between $O_{\text{seed}}$ and $O_{\text{truth},e}$. Since for every pair (*s_i_*, *s_j_*), $s_{i},s_{j}\in S$, we have computed the quantities $e_{s_{i},s_{j}}$ and (*t*, *k*), we output a pair (*t*, *k*) of values for every pair (*s_i_*, *s_j_*) such that $e_{s_{i},s_{j}}\geq e$, thus constructing $O_{\text{seed}}$.

As there could be many values of *k* satisfying $e_{s_{i},s_{j}}\geq e$, we would like to choose among them a relatively large value (see Section 1). Let $e_{\text{best}}$ be the highest alignment identity estimated over all considered *k* values for $s_{i},s_{j}\in S$, and $k_{\text{best}}$ be the *k* value corresponding to $e_{\text{best}}$. Further let *δ* be an optional input threshold parameter (with its default value set to 0.05). Then, we choose the maximum *k* value, which we denote by $k_{\delta }$, such that $e_{\text{best}}-e_{k_{\delta }}\leq \delta$, where $e_{k_{\delta }}$ is the alignment identity computed for $k=k_{\delta }$. We do that by iterating *k* over $\left[k_{\text{best}},k_{\text{max}}\right]$. The default value for *δ* and its usefulness is justified in the experiments.

In the next section, we show how the output of Seedability can be directly used to tune the alignment parameters of Minimap2.

## 4 Results

Seedability was implemented using the C++ programming language, taking in as input a set of sequences in multiFASTA format and an optional reference sequence in FASTA format. Seedability outputs optimal values for (*t*, *k*) either for the estimated alignment of all pairwise sequences or for the estimated alignment of the reference sequence and every sequence.

The source code is distributed under the GNU General Public License (GPL v3.0) at https://github.com/lorrainea/Seedability. We have conducted experiments on a computer using an Intel Core i5-8265U CPU, running at 1.60 GHz, equipped with 8GB of RAM, under GNU/Linux. Seedability was compiled with g++ version 9.3.0. Minimap2 (Li 2018) is a widely-used bioinformatics tool for aligning DNA or mRNA sequences to a large reference database. To evaluate the accuracy of Seedability, we applied the output values of Seedability on Minimap2 to check how alignment scores were impacted. This was carried out on both synthetic and real data.

### 4.1 Synthetic data

Synthetic data consisted of 100 pairs (*x*, *y*) of sequences with varying divergence threshold $d_{x,y}$, such that $0.05\leq d_{x,y}\leq 0.25$, and varying average length $\ell \in \{10^{2},200,300,500,10^{3},2000,5000,10^{4},15000\}$. The sequences were generated using the generate_dataset tool implemented as part of the Wavefront Alignment (WFA) tool (Marco-Sola *et al.* 2020).

Minimap2 has a wide range of preset options, which include default values for *κ* (the minimizer’s length) and *w* (the number of consecutive *κ*-mers considered for sampling). These preset options include:

1. map-ont—Align noisy long reads of ∼10% error rate to a reference sequence (default). (κ=15,w=10).
2. sr—Short single-end reads without splicing. (κ=21,w=11).
3. map-pb—Align older PacBio continuous long reads (CLR) to a reference sequence. (κ=19,w=12).
4. asm20—Long assembly to reference mapping (κ=19,w=10).

The average *k*-mer length output by Seedability, denoted by$\left\lceil k_{\text{avg}}\right\rceil$, was used to determine the (κ,w) values for Minimap2. We set $\kappa =\left\lceil k_{\text{avg}}\right\rceil$ and $w=\left\lceil \frac{2}{3}\kappa \right\rceil$. The value for *w* was determined using Mimimap2′s default value of $w=\frac{2}{3}\kappa$. Table 1 shows the determined (κ,w) values.

**Table 1.** The (κ,w) values determined by Seedability.

	Length								
Divergence	100	200	300	500	1000	2000	5000	10 000	15 000
0.05	(10,7)	(10,7)	(10,7)	(10,7)	(10,7)	(10,7)	(10,7)	(10,7)	(10,7)
0.10	(6,4)	(6,4)	(6,4)	(6,4)	(6,4)	(6,4)	(7,5)	(7,5)	(7,5)
0.15	(5,4)	(5,4)	(5,4)	(5,4)	(5,4)	(5,4)	(6,4)	(6,4)	(6,4)
0.20	(5,4)	(5,4)	(5,4)	(5,4)	(5,4)	(5,4)	(5,4)	(6,4)	(6,4)
0.25	(4,3)	(4,3)	(5,4)	(5,4)	(5,4)	(5,4)	(6,4)	(6,4)	(6,4)

**Figure 3.**
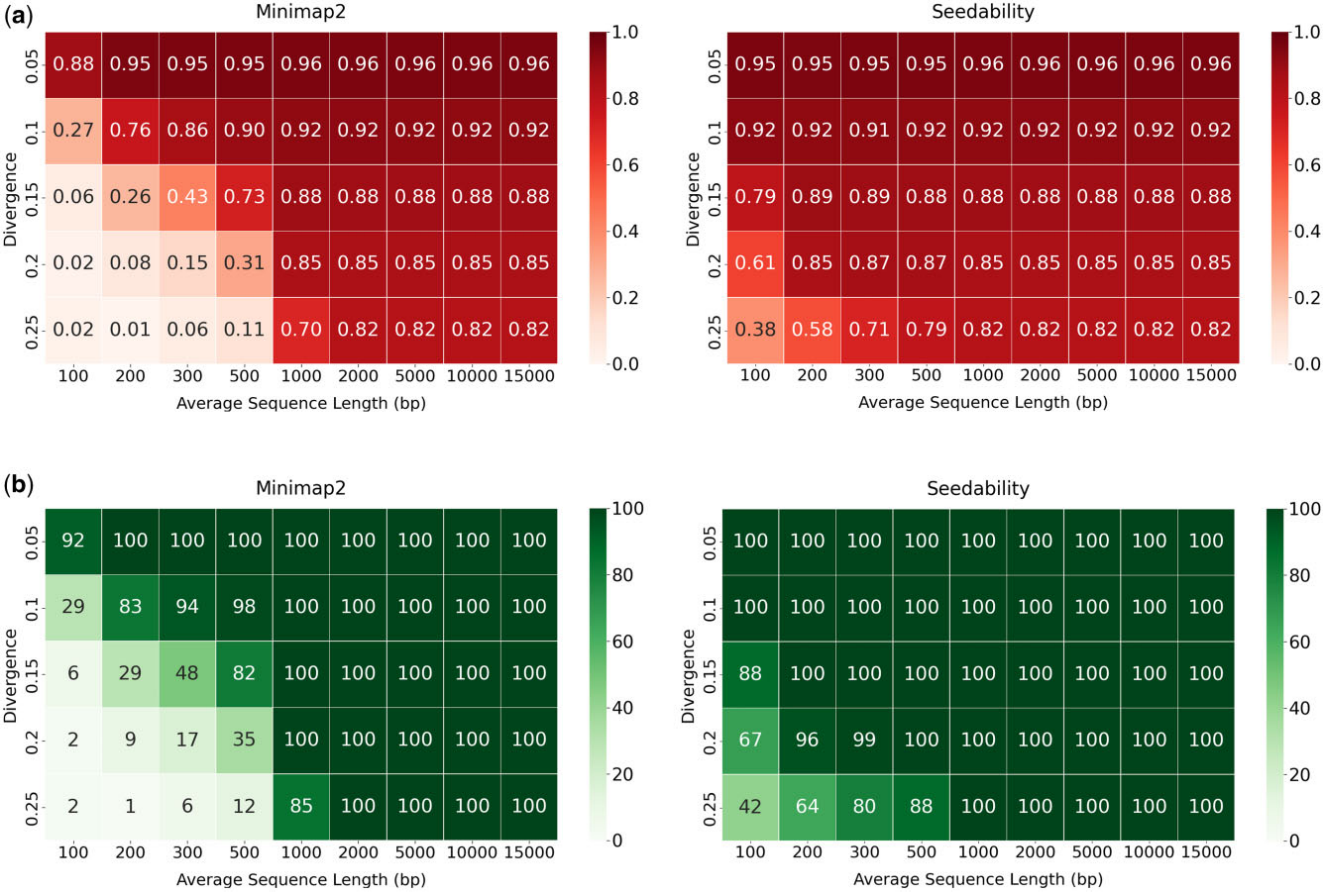
(a) The average alignment identities (i.e. the total alignment identity score divided by the total number of pairs) output for the 100 pairs of sequences when using the default Minimap2 (κ,w) values in comparison to the (κ,w) values determined by Seedability. For the preset options, we have used: (i) the default preset option map-ont, if the average sequence length is ≥1000; or (ii) the preset option sr, if the average sequence length is <1000. (b) The number of mapped alignments when using the default Minimap2  (κ,w) values in comparison to the (κ,w) values determined by Seedability. For the preset options, we have used: (i) the default preset option map-ont, if the average sequence length is ≥1000; or (ii) the preset option sr, if the average sequence length is <1000.

**Figure 4.**
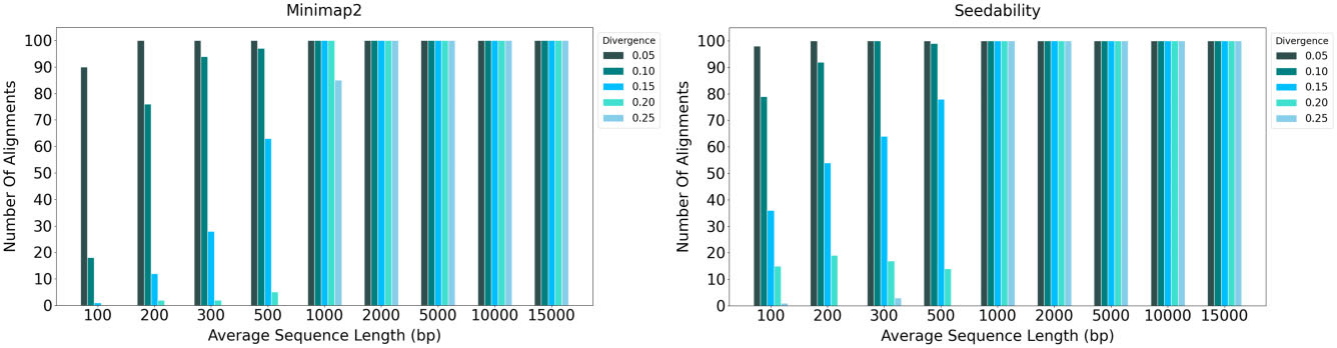
The number of alignments that have an alignment length at least 90% of the original sequence length when using the default Minimap2(κ,w) values in comparison to the (κ,w) values determined by Seedability. For the preset options, we have used: (i) the default preset option map-ont, if the average sequence length is ≥1000; or (ii) the preset option sr, if the average sequence length is <1000.

Figure 3a shows the average alignment identities (i.e. the total alignment identity score divided by the total number of pairs) output for the 100 pairs of sequences when using the default (κ,w) values in comparison to the (κ,w) values determined by Seedability. For the preset options we used: (i) the default preset option map-ont, if the average sequence length is greater or equal to 1000; or (ii) the preset option sr, if the average sequence length is <1000. The parameter values produced by Seedability allow Minimap2 to maintain high alignment identities for longer sequences but also vastly improve the alignment identities for shorter sequences. Note that some of these alignments were unmapped with Figure 3b showing the number of mapped alignments identified. Further, note that the alignment identities computed by Minimap2 were higher than the expected identities due to the mapping quality of Minimap2. The alignment identities are computed as a fraction of the number of matching bases over the total number of bases, including gaps as defined by Minimap2. Figure 4 shows the number of alignments produced out of the 100 pairs of sequences. In this case, a pair of sequences are said to be aligned if the alignment length is at least 90% of the original sequence length. It is specifically clear that for all sequences aligned using preset option sr, the parameter values determined by Seedability resulted in the output of improved alignments. The Supplementary Material shows analogous experimental results for other preset options. These results already establish the usefulness of Seedability.

As previously mentioned, Seedability also estimates the number *t* of shared seeds, for an output *k* value, that can be found within an aligned pair of sequences. Table 2 shows the number of pairs of sequences out of 100 where *t* is within ±10% of the number of seeds in an optimal alignment. We set *δ *= 0 to evaluate how well Seedability can perform this task.

**Table 2.** The number of sequence pairs where *t* output by Seedability, for the output *k* value, is within ±10% of the number of seeds in an optimal alignment.

	Length								
Divergence	100	200	300	500	1000	2000	5000	10 000	15 000
0.05	100	100	100	100	100	100	100	100	100
0.10	100	100	100	100	100	100	100	100	100
0.15	100	100	100	100	100	100	100	100	100
0.20	100	100	100	100	98	100	98	97	94
0.25	100	99	100	98	98	92	93	84	89

To evaluate the symmetric difference between $O_{\text{seed}}$ and $O_{\text{truth},e}$, we counted the number of alignments computed by Seedability, which have an estimated alignment identity $e_{\text{best}}\geq e$. We created two datasets both containing 200 pairs of sequences: the first one consisted of sequences with an average length of 200; and the second one with an average length of 500. Both datasets contained 100 pairs of sequences with a divergence of 0.10 and 100 pairs of sequences with a divergence of 0.20. Table 3 shows the number of true positives (TP), false positives (FP), true negatives (TN) and false negatives (FN) identified within each dataset. When *e *=* *0.85 and the average length of the sequences is 200, Seedability was able to identify 98 out of 100 pairs of sequences such that $e_{\text{best}}\geq e$. For the same *e* and when the average length of the sequences is 500, Seedability was able to identify 100 out of 100 pairs of sequences such that $e_{\text{best}}\geq e$. In this second case $O_{\text{seed}}=O_{\text{truth},e}$. The results show that, although Seedability underestimates alignment identity by a little—which is expected as it is not meant to compute optimal alignments—it minimizes the symmetric difference of $O_{\text{seed}}$ and $O_{\text{truth},e}$ by computing appropriate (*t*, *k*) values. Furthermore, these results justify the existence of *δ* and its default value.

**Table 3.** The number of true positives (TP), false positives (FP), true negatives (TN) and false negatives (FN) reported by Seedability ($e_{\text{best}}\geq e$) when looking at 100 alignments with a divergence of 0.10 and 100 with a divergence of 0.20

	Length							
	200				500			
*e*	TP	FP	TN	FN	TP	FP	TN	FN
0.90	47	0	100	53	33	0	100	67
0.89	67	0	100	33	60	0	100	40
0.88	82	0	100	18	88	0	100	12
0.87	93	0	100	7	94	0	100	6
0.86	96	0	100	4	97	0	100	3
0.85	98	2	98	2	100	0	100	0

**Figure 5.**
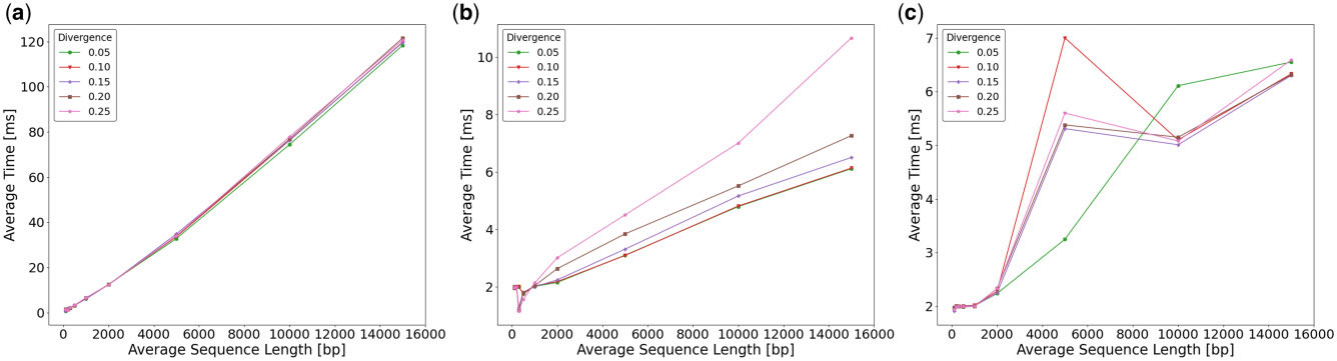
The average time in ms required when using preset option map-ont for (a) Seedability to compute (*t*, *k*), (b) Minimap2 to compute an alignment using default parameter values, and (c) Minimap2 to compute an alignment using the (κ,w) values determined by Seedability.

**Figure 6.**
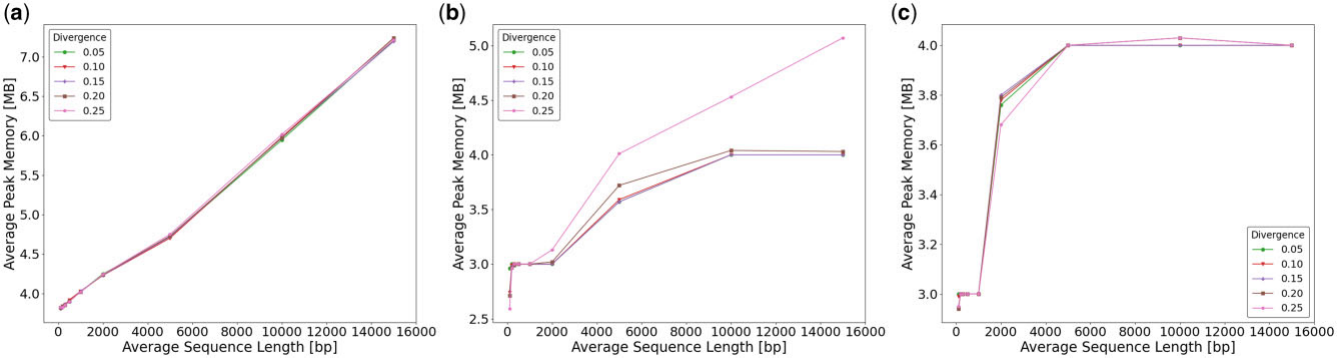
The average peak memory in MB required when using preset option map-ont for (a) Seedability to compute (*t*, *k*), (b) Minimap2 to compute an alignment using default parameter values and (c) Minimap2 to compute an alignment using the (κ,w) values determined by Seedability.

Figure 5a shows the average time required by Seedability to compute (*t*, *k*). Figure 5b shows the average time required by Minimap2 to compute an alignment when using its default values and Fig. 5c shows likewise when using the values determined by Seedability. Figure 6 shows the same but for the average peak memory. Recall from Section 3.2 that the whole computation of Seedability takes $O(k(|s_{i}|+|s_{j}|))$ time for any pair $s_{i},s_{j}\in S$ of sequences and any $k\in [\min_{k},\max_{k}]$. It is also clear from the results (Figs 5a and 6a) that Seedability requires linear time and space for the values of *k* used in practice. For divergence <0.25, Minimap2 takes a similar time when using the values determined by Seedability in comparison to when using its default values. Notably, for divergence 0.25, Minimap2 is faster when using the values determined by Seedability. When using the values determined by Seedability, Minimap2 uses similar peak memory as when its default parameter values are used. In these experiments, we have used the preset option map-ont. The Supplementary Material shows similar results for the other preset options.

### 4.2 Real data

To further highlight the usefulness of Seedability, we have considered real data. We looked at a Chimpanzee gene, in particular, gene ENSPTRG00000044036 (125 bp in length) as well as the orthologues of this gene with the following species: the Algerian Mouse (*Mus spretus*) (126 bp in length); the Northern American deer mouse (*Peromyscus maniculatus bairdii*) (125 bp in length); and the Shrew mouse (*Mus pahari*) (114 bp in length). These sequences have an optimal alignment identity of 0.744, 0.752, and 0.729, respectively. The orthologue identities were retrieved from the Ensebl genome browser (Howe *et al.* 2020). We used the preset option sr and default (κ,w) values of Minimap2 to align the sequences. There were *no output alignments* for the three pairs of sequences. Seedability was then used to identify optimal (κ,w) parameter values for Minimap2. The computed output values were (6, 4), (5, 4), and (5, 4), respectively, for the listed orthologues. The sequence pairs were re-aligned using the (κ,w) values determined by Seedability and the resulting alignment identities computed were 0.857, 0.845, and 0.895, respectively. Note that the alignment identities computed by Minimap2 were higher than the original identities due to the mapping quality of Minimap2.

**Figure 7.**
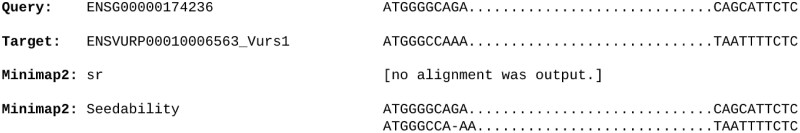
Human gene versus ortholog alignment produced by Minimap2 when using (κ=4,w=3) determined by Seedability in comparison to *no output alignment* produced when using the default (κ,w) values of Minimap2.

We also carried out similar experiments for the RAB15EP gene in human chromosome 12 (ENSG00000174236) (708 bp in length) with orthologues of this gene with the following species: the Abingdon island giant tortoise (*Chelonoidis abingdonii*) (699 bp in length); the Argentine black and white tegu (*Salvator merianae*) (714 bp in length); and the Common wombat (*Vombatus ursinus*) (708 bp in length). These sequences have an optimal alignment identity of 0.619, 0.567, and 0.538, respectively. There were again *no output alignments* by Minimap2 for the three pairs of sequences. Seedability was then used to identify optimal (κ,w) values for Minimap2 which were computed to be (4, 3), (3, 2), and (4, 3), respectively for the listed orthologues. The sequence pairs were re-aligned using the (κ,w) values determined by Seedability and the resulting alignment identities were 0.686, 0.635, and 0.734, respectively. Figure 7 shows a visual representation of the results for the alignment of ENSG00000174236 and ENSVURP00010006563_Vurs1 (*Vombatus ursinus*).

**Figure 8.**
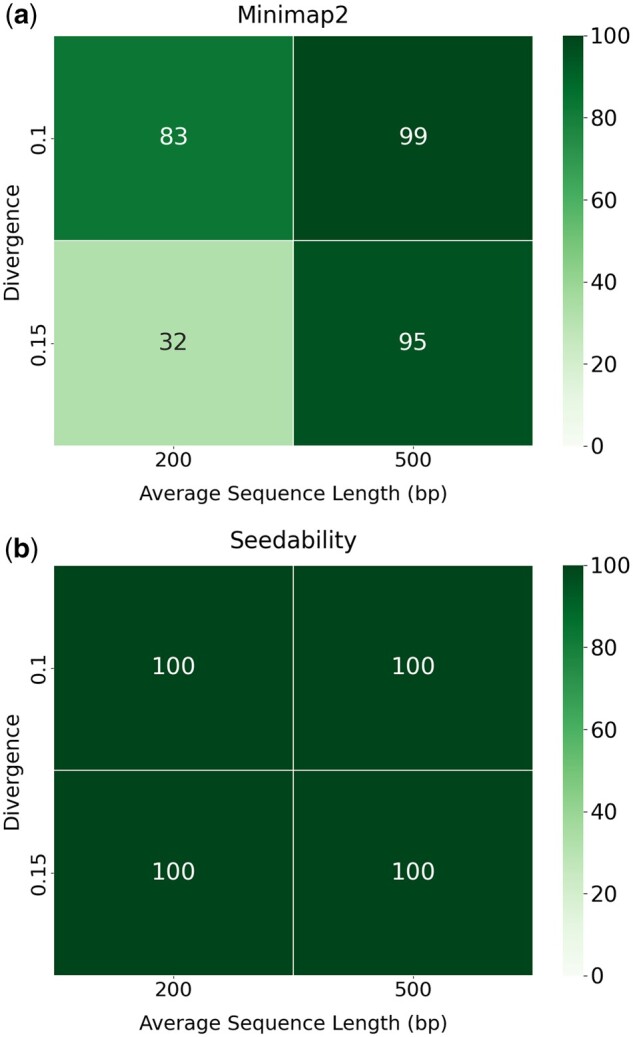
(a) The number of mapped sequences when using the default (κ,w) values of Minimap2. (b) The number of mapped sequences when using the (κ,w) values determined by Seedability from Table 1.

The results produced in Table 1 can be used directly to improve the alignment identities of short sequences when mapping to predetermined candidate positions on a reference genome. We tested the (κ,w) values presented in Table 1 on simulated reads from Chromosome 1 of the human genome (version GRCh38.p14). We used PBsim (Ono *et al.* 2020), a sequence simulator, to generate four datasets using GRCh38.p14: one dataset with an average length of 200 and divergence 0.10; one dataset with an average length of 200 and divergence 0.15; one dataset with an average length of 500 and divergence 0.10; and one dataset with an average length of 500 and divergence 0.15. Pairs of sequences were created by taking each simulated read and its original sequence interval in the genome. All datasets contained 100 pairs of sequences. Figure 8a shows the number of sequences out of 100 that were mapped when using the default (κ,w) values for Minimap2. Figure 8b shows the number of sequences out of 100 that were aligned when using the (κ,w) values determined by Seedability in Table 1. Note that for all experiments, the default preset map-ont was used. The parameter values determined by Seedability were able to produce mapped alignments for all sequences unlike when using the default parameter values of Minimap2. In particular, when using a divergence of 0.15 and length 200, the default parameter values of Minimap2 resulted in *only 32 mapped alignments*, that is, 68 fewer than when using the parameter values determined by Seedability.

Table 4 shows the average time in ms required to map the 100 sequences to candidate positions in Chromosome 1 of the human genome when using Minimap2’s default (*κ*,*w*) values in comparison to those determined by Seedability. The datasets are presented in the table in the form A.B where A is the average length of the sequences and B is their divergence. The difference in time between the two runs is negligible. In fact, for the datasets with an average length of 500, Minimap2 performed faster when using the parameter values determined by Seedability in comparison to the default parameter values. Table 5 shows similar results for the peak memory required to compute the mappings. Again it is clear that for the datasets with an average length of 500, Minimap2 used, on average, less peak memory when using the parameter values determined by Seedability in comparison to the default parameter values.

**Table 4.** The average time in ms required to map 100 sequences to candidate positions in Chromosome 1 of the human genome when using Minimap2’s default (*κ*,*w*) values in comparison to those determined by Seedability.

	200.10	200.15	500.10	500.15
Minimap2	1.21	1.19	1.97	2.14
Seedability	1.03	1.05	1.09	1.28

**Table 5.** The average peak memory in MB required to map 100 sequences to candidate positions in Chromosome 1 of the human genome when using Minimap2’s default (*κ*,*w*) values in comparison to those determined by Seedability.

	200.10	200.15	500.10	500.15
Minimap2	2.86	2.80	3.06	3.05
Seedability	2.87	2.89	2.92	2.91

## 5 Discussion

A large number of existing bioinformatics tools aim to perform sensitive sequence comparisons. Winnowmap2 (Jain *et al.* 2022) is based on Minimap2 but does not use fixed-length *k*-mers as seeds. NGMLR (Sedlazeck *et al.* 2018) is designed to sensitively align PacBio or Oxford Nanopore reads to large reference genomes for structural variant calling. In many practical scenarios, identifying optimal *k* values is challenging, and default *k* values provide suboptimal results.

In this article, we presented Seedability, an alignment framework designed for estimating an optimal value for *k* as well as a minimal number *t* of shared seeds based on a given alignment identity threshold. Our extensive results, using both synthetic and real datasets, demonstrate that the (*t*, *k*) values determined by Seedability lead to improved alignments compared to the original alignments produced by Minimap2 when using sequences with lengths of a varying range and a varied divergence. Notably, the parameter values determined by Seedability lead to meaningful alignments in some cases where *no output alignments* were produced using the default parameter values of Minimap2.

For future work, we would be interested in extending Seedability to support BLEND (Firtina *et al.* 2023), which hashes seeds to identify similarities between sequences as well as extending Seedability to support mapquik (Ekim *et al.* 2023), a tool that makes use of longer seeds through matches of *k* consecutively sampled minimizers.

## Supplementary Material

vbad108_Supplementary_Data

## Data Availability

The data underlying this article are available either in https://github.com/lorrainea/Seedability or in the ensembl database at www.ensembl.org, and can be accessed using the gene names ENSPTRG00000044036 and ENSG00000174236 or in the NCBI database at www.ncbi.nlm.nih.gov and can be found using the reference sequence NC_000001.11.
