# Supplementary material for: Seedability: optimizing alignment parameters for sensitive sequence comparison
Source: Bioinform Adv. 2023 Aug 12;3(1):vbad108. doi: 10.1093/bioadv/vbad108 (PMC10444664; doi:10.1093/bioadv/vbad108)
Supplement: vbad108_Supplementary_Data [file vbad108_supplementary_data.pdf]

Supplement to "Seedability: Optimising alignment parameters for sensitive sequence comparison"

In Section 1 we give more results on sensitivity using synthetic data for different preset options of Minimap2. In Section 2 we give more results on efficiency using synthetic data for the same options.

1 Sensitivity on Synthetic Data

1.1 Preset Option `map-ont`

Figure 1a shows the average alignment identities (i.e., the total alignment identity score divided by the total number of pairs) output for the 100 pairs of sequences when using the default (κ, w) values in comparison to the (κ, w) values determined by Seedability. Figure 1b shows the number of mapped alignments. The preset option `map-ont` was used. It is clear that for all datasets, the parameter values determined by Seedability resulted in the output of more sensitive alignments. It can also be seen that the parameter values determined by Seedability allowed Minimap2 to produce an alignment for all pairs in most datasets, which is not the case when the default parameter values were used.

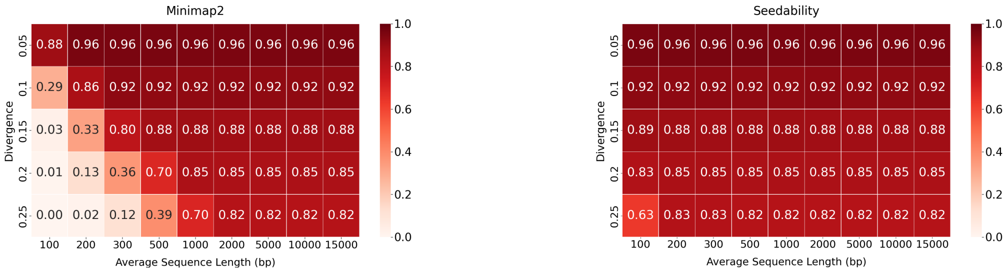

(a) The average alignment identities (i.e., the total alignment identity score divided by the total number of pairs) output for the 100 pairs of sequences when using the default Minimap2 (κ, w) values in comparison to the (κ, w) values determined by Seedability. The preset option `map-ont` was used.

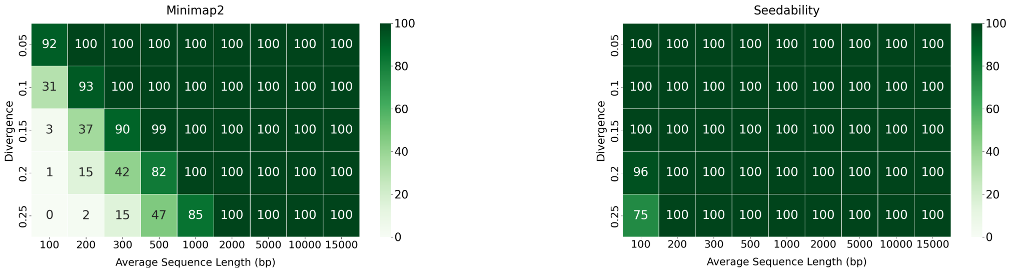

(b) The number of mapped alignments when using the default Minimap2 (κ, w) values in comparison to the (κ, w) values determined by Seedability. The preset option `map-ont` was used.

Figure 1

Figure 2 shows the number of alignments that have an alignment length at least 90% of the original sequence length using the default (κ, w) values in comparison to the (κ, w) values determined by Seedability. The preset option `map-ont` was used. It is clear that Minimap2 produces more sensitive alignments when using the parameter values determined by Seedability.

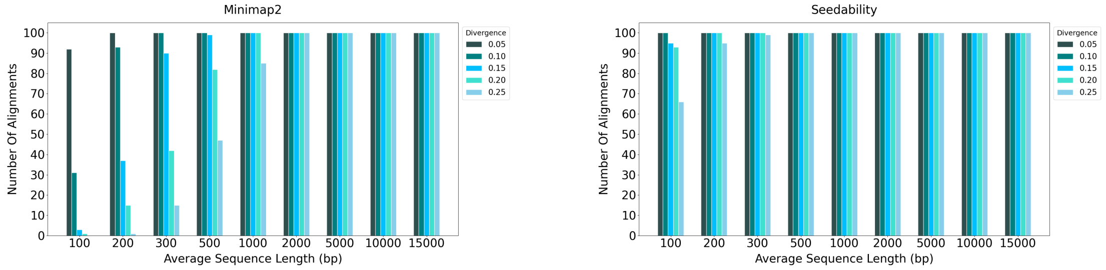

Figure 2: The number of alignments that have an alignment length at least 90% of the original sequence length when using the default Minimap2 (κ, w) values in comparison to the (κ, w) values determined by Seedability. The preset option `map-ont` was used.

1.2 Preset Option `map-pb`

Figures 3 and 4 show the analogous experiments for preset option `map-pb`. It is clear that Minimap2 produces more sensitive alignments when using the parameter values determined by Seedability.

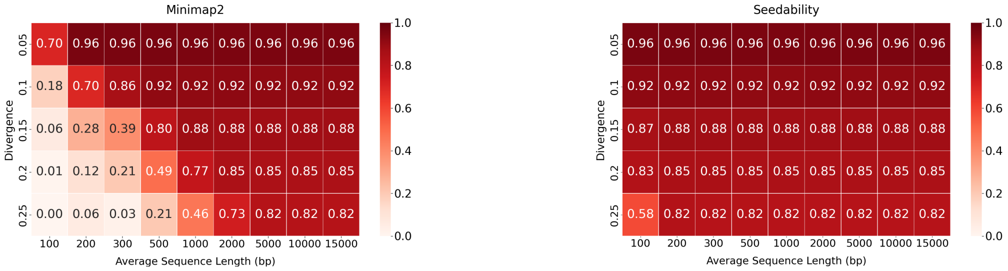

(a) The average alignment identities (i.e., the total alignment identity score divided by the total number of pairs) output for the 100 pairs of sequences when using the default Minimap2 (κ, w) values in comparison to the (κ, w) values determined by Seedability. The preset option `map-pb` was used.

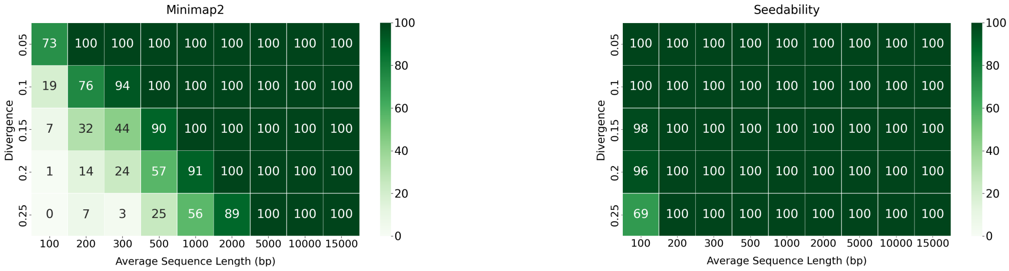

(b) The number of mapped alignments when using the default Minimap2 (κ, w) values in comparison to the (κ, w) values determined by Seedability. The preset option `map-pb` was used.

Figure 3

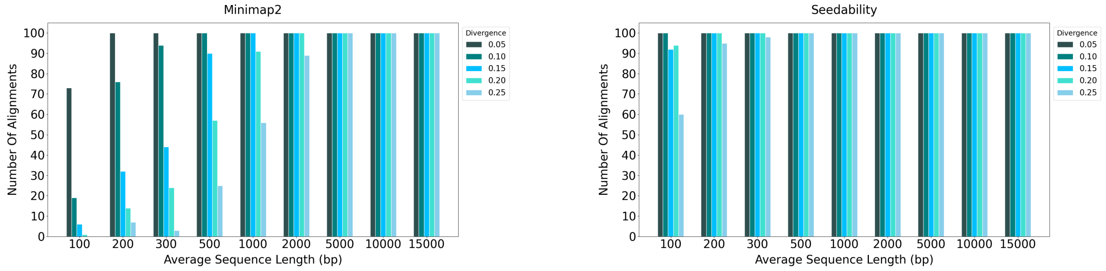

Figure 4: The number of alignments that have an alignment length at least 90% of the original sequence length when using the default Minimap2 (κ, w) values in comparison to the (κ, w) values determined by Seedability. The preset option `map-pb` was used.

1.3 Preset Option asm20

Figures 5 and 6 show the analogous experiments for preset option asm20. When looking at Figure 5, it is clear that Minimap2 produces the same or slightly more sensitive alignments when using its default parameter values. From Figure 6, it is clear that Minimap2 computes the same length or longer alignments when using the parameters determined by Seedability.

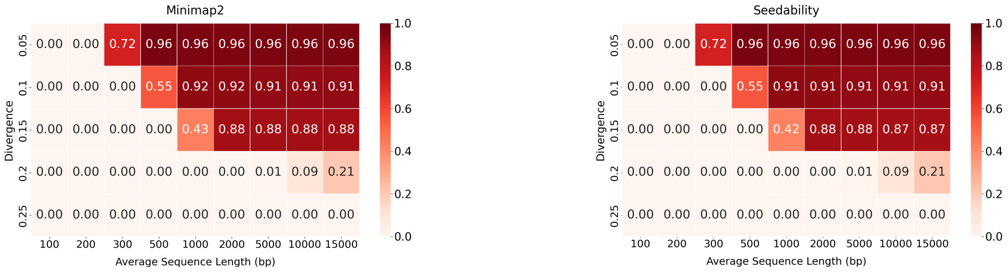

(a) The average alignment identities (i.e., the total alignment identity score divided by the total number of pairs) output for the 100 pairs of sequences when using the default Minimap2 (κ, w) values in comparison to the (κ, w) values determined by Seedability. The preset option asm20 was used.

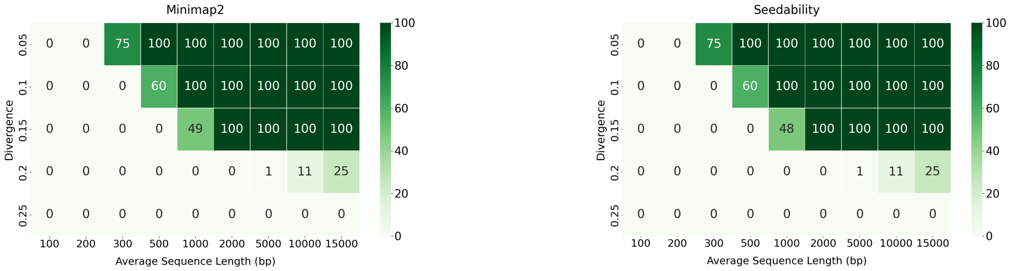

(b) The number of mapped alignments when using the default Minimap2 (κ, w) values in comparison to the (κ, w) values determined by Seedability. The preset option asm20 was used.

Figure 5

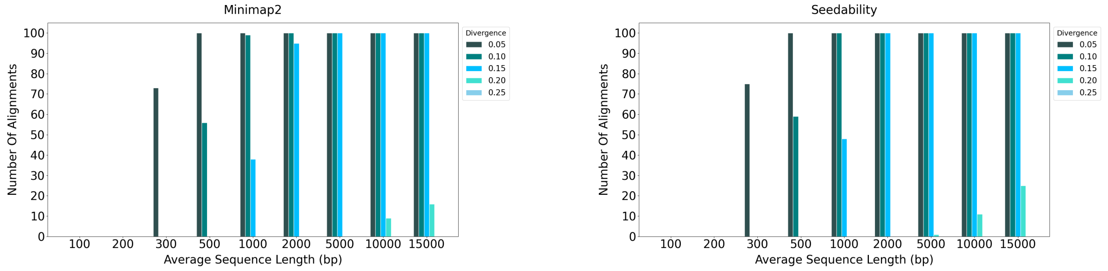

Figure 6: The number of alignments that have an alignment length at least 90% of the original sequence length when using the default Minimap2 (κ, w) values in comparison to the (κ, w) values determined by Seedability. The preset option asm20 was used.

1.4 Preset Option sr

Figures 7 and 8 show the analogous experiments for preset option sr. As depicted in Figure 7, for $\ell \leq 500$, the parameter values determined by Seedability produce more sensitive alignments than when using the default parameter values of Minimap2. Notice that the alignment result produced by Minimap2 for $\ell = 15000$ and $d_{x,y} = 0.25$ seems to be significantly larger than the optimal alignment identity of the dataset, showing that there are inaccuracies in the alignments produced by Minimap2 when using preset option sr. It should, however, be noted that preset option sr has been designed for short reads.

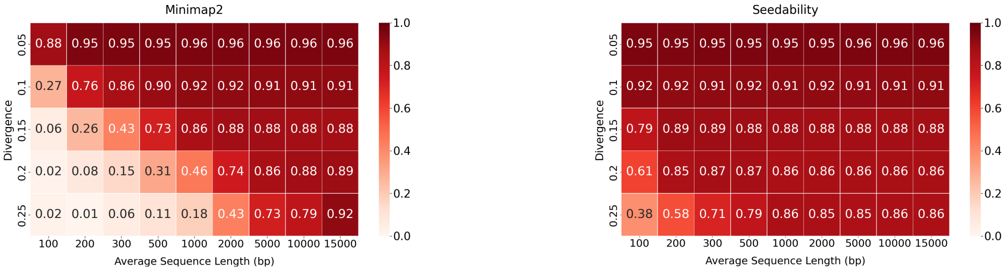

(a) The average alignment identities (i.e., the total alignment identity score divided by the total number of pairs) output for the 100 pairs of sequences when using the default Minimap2 (κ, w) values in comparison to the (κ, w) values determined by Seedability. The preset option sr was used.

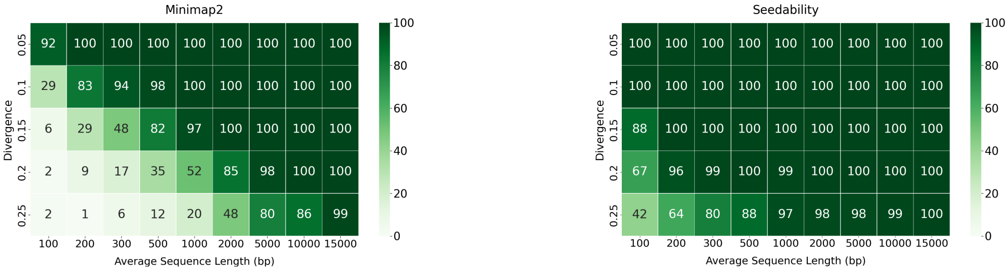

(b) The number of mapped alignments when using the default Minimap2 (κ, w) values in comparison to the (κ, w) values determined by Seedability. The preset option sr was used

Figure 7

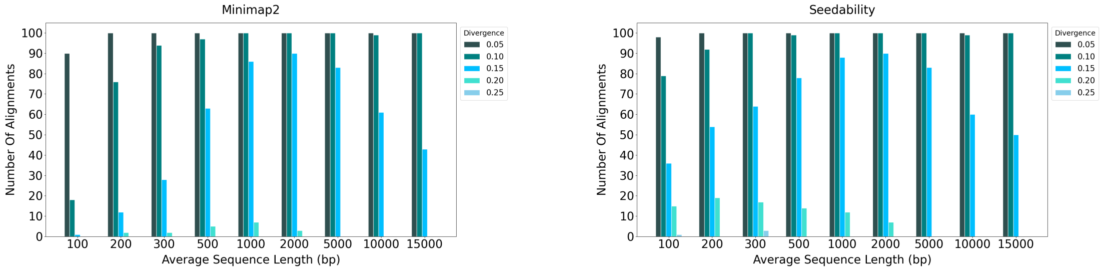

Figure 8: The number of alignments that have an alignment length at least 90% of the original sequence length when using the default Minimap2 (κ, w) values in comparison to the (κ, w) values determined by Seedability. The preset option sr was used.

2 Efficiency on Synthetic Data

2.1 Preset Option `map-pb`

Figure 9a shows the average time required by Minimap2 to compute an alignment when using its default parameter values and preset option `map-pb` and Figure 9b shows likewise when using the values determined by Seedability and preset option `map-pb`. Figure 10 shows the same but for the average peak memory. Minimap2 takes approximately the same amount of time when using the default parameter values or the values determined by Seedability for sequences with a divergence less than 0.20. As the divergence and average sequence length increase, Minimap2 becomes faster when using the values determined by Seedability compared to its default parameter values. For divergences less than 0.20, when using the values determined by Seedability, Minimap2 uses approximately the same peak memory as when the default parameter values are used. For divergences 0.20 and 0.25, Minimap2 uses less peak memory when the values determined by Seedability are used.

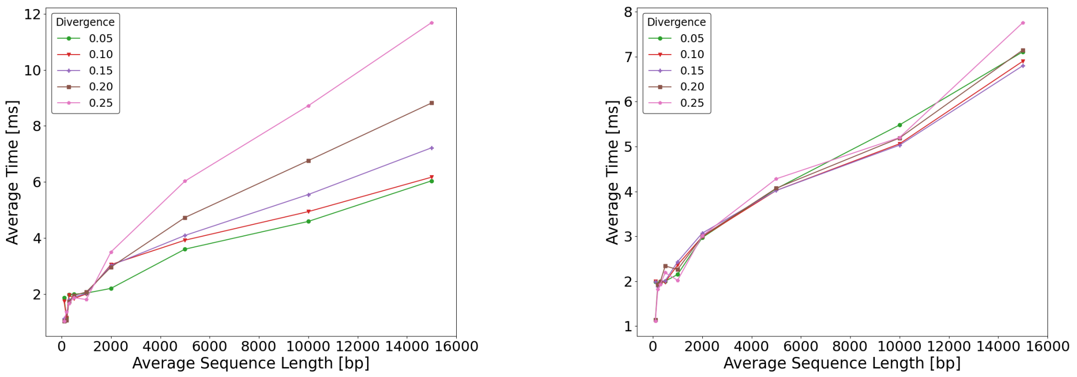

(a) (b)

Figure 9: The average time in ms required when using preset option `map-pb` for (a) Minimap2 to compute an alignment using default parameter values and (b) Minimap2 to compute an alignment using the (κ, w) values determined by Seedability.

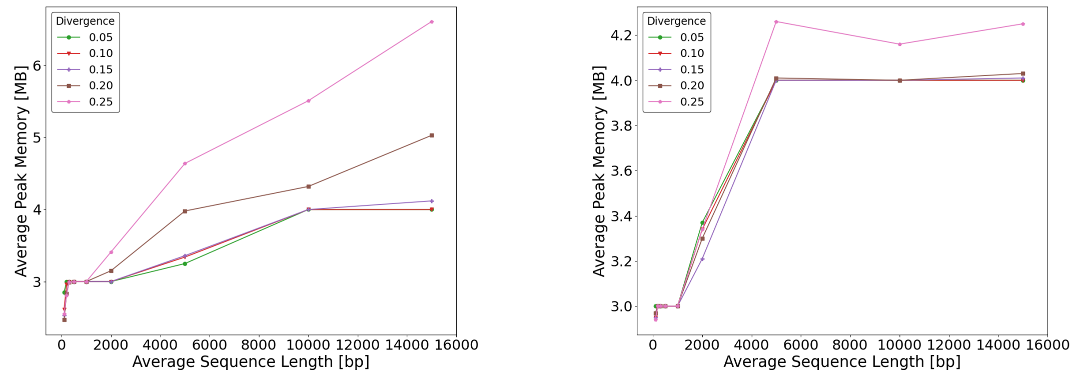

(a) (b)

Figure 10: The average peak memory in MB required when using preset option `map-pb` for (a) Minimap2 to compute an alignment using default parameter values and (b) Minimap2 to compute an alignment using the (κ, w) values determined by Seedability.

2.2 Preset Option asm20

Figure 11a shows the average time required by Minimap2 to compute an alignment when using its default parameter values and preset option asm20 and Figure 11b shows likewise when using the values determined by Seedability and preset option asm20. Figure 12 shows the same but for the average peak memory. As the average sequence length increases, Minimap2 is faster for divergences 0.25 and 0.20 when using the parameters determined by Seedability. For divergences less than 0.20, when using the values determined by Seedability, Minimap2 uses approximately the same peak memory as when the default parameter values are used. For divergences 0.20 and 0.25, Minimap2 uses less peak memory when the values determined by Seedability are used.

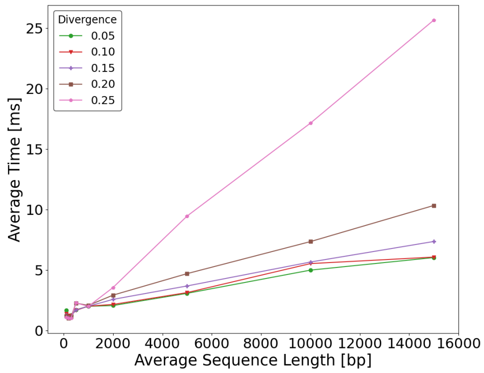

(a)

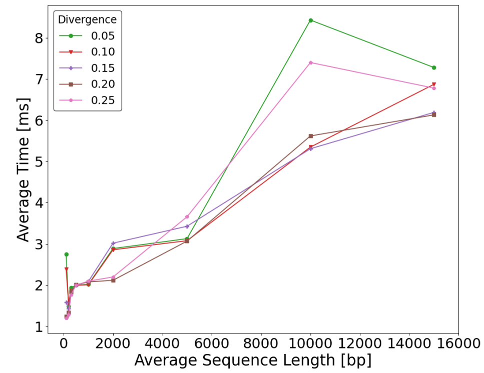

(b)

Figure 11: The average time in ms required when using preset option asm20 for (a) Minimap2 to compute an alignment using default parameter values and (b) Minimap2 to compute an alignment using the (κ, w) values determined by Seedability.

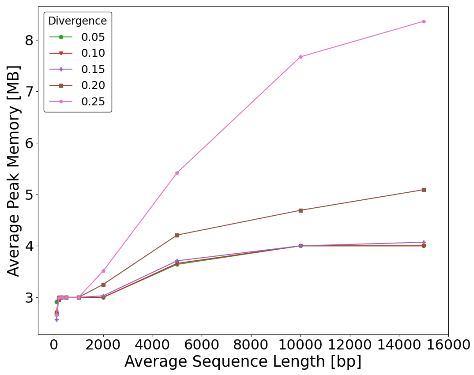

(a)

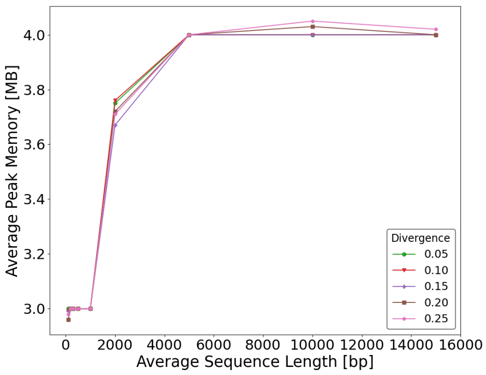

(b)

Figure 12: The average peak memory in MB required when using preset option asm20 for (a) Minimap2 to compute an alignment using default parameter values and (b) Minimap2 to compute an alignment using the (κ, w) values determined by Seedability.

2.3 Preset Option sr

Figure 13a shows the average time required by Minimap2 to compute an alignment when using its default parameter values and preset option `sr`; and Figure 13b shows likewise when using the values determined by Seedability and preset option `sr`. Figure 14 shows the same but for the average peak memory. For divergences less than 0.20, Minimap2 is up to 4 times faster when using the (κ, w) values determined by Seedability in comparison to the default parameter values. Minimap2 uses approximately the same or slightly more peak memory when using the (κ, w) values determined by Seedability in comparison to when the default parameter values are used.

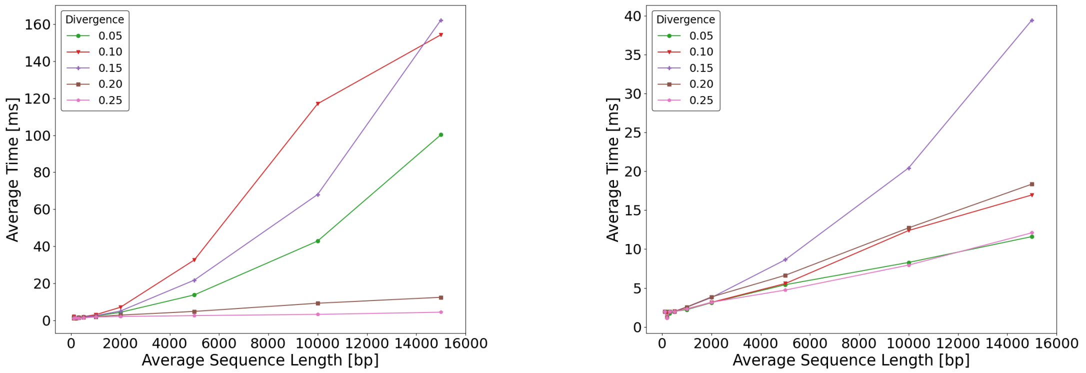

(a) (b)

Figure 13: The average time in ms required when using preset option `sr` for (a) Minimap2 to compute an alignment using default parameter values and (b) Minimap2 to compute an alignment using the (κ, w) values determined by Seedability.

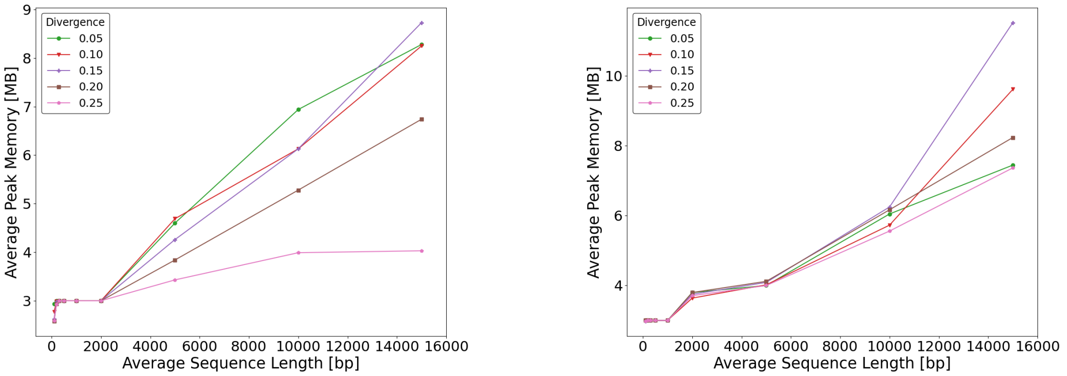

(a) (b)

Figure 14: The average peak memory in MB required when using preset option `sr` for (a) Minimap2 to compute an alignment using default parameter values and (b) Minimap2 to compute an alignment using the (κ, w) values determined by Seedability.
